# Impact and Sustainability of Antibiotic Stewardship in Pediatric Emergency Departments: Why Persistence Is the Key to Success

**DOI:** 10.3390/antibiotics9120867

**Published:** 2020-12-04

**Authors:** Elisa Barbieri, Maia De Luca, Marta Minute, Carmen D’Amore, Marta Luisa Ciofi Degli Atti, Stefano Martelossi, Carlo Giaquinto, Liviana Da Dalt, Theoklis Zaoutis, Daniele Dona

**Affiliations:** 1 Division of Pediatric Infectious Diseases, Department of Women’s and Children’s Health, University of Padova, 35131 Padova, Italy; carlo.giaquinto@unipd.it (C.G.); daniele.dona@unipd.it (D.D.); 2 Unit of Immune and Infectious Diseases, Academic Department of Pediatrics, Bambino Gesù Children’s Hospital, IRCCS, 00165 Rome, Italy; maia.deluca@opbg.net; 3 Pediatric Unit, Ca’ Foncello’s Hospital, 31100 Treviso, Italy; marta.minute@aulss2.veneto.it (M.M.); stefano.martelossi@aulss2.veneto.it (S.M.); 4 Unit of Clinical Epidemiology, Bambino Gesù Children’s Hospital, IRCCS, 00165 Rome, Italy; carmen.damore@opbg.net (C.D.); marta.ciofidegliatti@opbg.net (M.L.C.D.A.); 5 Pediatric Emergency Department, Department of Women’s and Children’s Health, University Hospital of Padua, 2-35128 Padova, Italy; liviana.dadalt@unipd.it; 6 Division of Infectious Diseases and the Center for Pediatric Clinical Effectiveness, Children’s Hospital of Philadelphia, Philadelphia, PA 19104, USA; zaoutis@email.chop.edu

**Keywords:** antibiotic stewardship, pharyngitis, acute otitis media, clinical pathways, children, emergency departments, antibiotic use, prescribing appropriateness

## Abstract

Antibiotic stewardship programs proved to be effective in improving prescribing appropriateness. This multicenter quasi-experimental study, aimed to assesses the stewardship impact on antibiotics prescribing in different semesters from 2014 to 2019 in three pediatric emergency departments (Center A, B, and C) in Italy. All consecutive patients diagnosed with acute otitis media or pharyngitis were evaluated for inclusion. Two different stewardship were adopted: for Center A and B, clinical pathways were implemented and disseminated, and yearly lectures were held, for Center C, only pathways were implemented. Broad-spectrum prescription rates decreased significantly by 80% for pharyngitis and 29.5 to 55.2% for otitis after the implementation. In Center C, rates gradually increased from the year after the implementation. Amoxicillin dosage adjusted to pharyngitis recommendations in Center C (53.7 vs. 51.6 mg/kg/die; *p* = 0.011) and otitis recommendations in Center A increasing from 50.0 to 75.0 mg/kg/die (*p* < 0.001). Days of therapy in children < 24 months with otitis increased from 8.0 to 10.0 in Center A, while in older children decreased in Center A (8.0 vs. 7.0; *p* < 0.001) and Center B (10.0 vs. 8.0; *p* < 0.001). Clinical pathways combined with educational lectures is a feasible and sustainable program in reducing broad-spectrum antibiotic prescribing with stable rates over time.

## 1. Introduction

Antibiotics remain the most commonly prescribed drugs in the pediatric population [1], with pharyngitis and acute otitis media (AOM) accounting for more than half of the prescriptions in the emergency departments and primary care practices [2,3], with an overprescribing of broad-spectrum antibiotics.

Both conditions have a viral and a bacterial etiology: AOM is mostly caused by Streptococcus pneumoniae, non-typeable *Haemophilus influenzae*, and *Moraxella catarrhalis* [4], while around 20% of pharyngitis are caused by Group A β-hemolytic streptococcus [5].

Although most of the pathogens remain sensible to first line amoxicillin, co-amoxiclav and III-gen cephalosporins are, respectively, prescribed in around 30% and 15% of AOM primary care cases, and in more than 24% and 15% of Group A streptococcus pharyngitis cases [3].

To decrease or reverse this trend, various antibiotic stewardship programs (ASPs) have been implemented worldwide, focusing on different approaches [6].

Clinical pathways have proven to be a feasible and efficient first step in improving prescribing appropriateness, especially in settings where funding is limited [7,8,9,10]. A clinical pathway is a task-oriented plan designed to support the implementation of clinical guidelines and protocols in primary care and inpatient settings.

In October 2015, an ASP based on clinical pathways was implemented in the pediatric emergency department of Padova University Hospital. Preliminary results reported an increase in “wait-and-see” approach rate for AOM (21.7% vs. 33.1%) and an increase in narrow-spectrum antibiotics treatments for both AOM (32.0% vs. 51.6%) and pharyngitis (53.6% vs. 93.4%), with no variation in treatment failures [10].

While there is not yet a consensus on the most effective ASP—especially in terms of settings and costs—we aimed to evaluate the efficacy and sustainability over time of ASPs based on clinical pathways with and without yearly educational lectures in three pediatric emergency departments.

## 2. Results

### 2.1. Pharyngitis

During the study, 4534 pharyngitis episodes were evaluated, accounting for around 3% of total pediatric emergency department visits. In total, 3249 episodes were included; the demographic characteristics of children included were similar with respect to sex, with a higher prevalence among older children in both Center B and C (Supplementary Materials, Table S2).

In Center A and B amoxicillin prescriptions rate increased (Center A: from 53.6% to 98.5%; *p* < 0.001, Center B: from 69.7% to 96.0%, *p* < 0.001) with a consequent decrease in broad-spectrum-antibiotic prescription rates (broad-spectrum antibiotic prescriptions) (Table 1).

**Figure 1:**
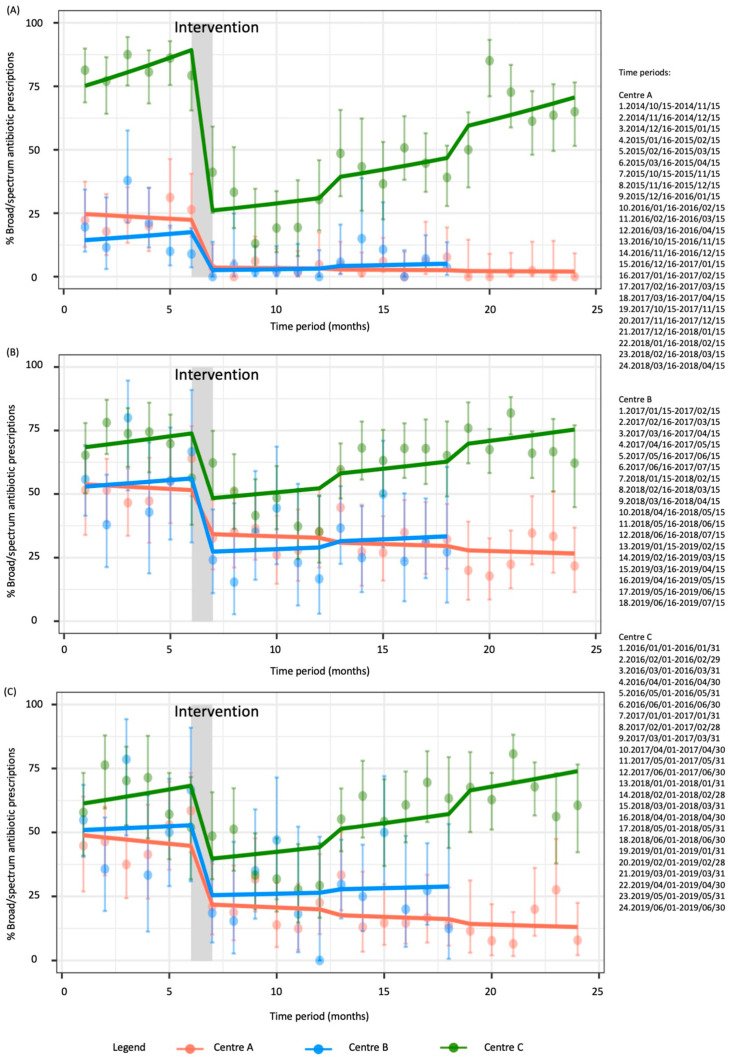
Interrupted time series of monthly broad-spectrum antibiotics prescriptions (dots) expressed as percentages with 95% confidence intervals (bars) for (**A**) pharyngitis, (**B**) acute otitis media, and (**C**) non-complicated acute otitis media in the three centers. The lines represent the broad-spectrum prescriptions trend in the different centers.

The interrupted time series model strongly suggest a broad-spectrum antibiotic prescriptions reduction following the introduction of the clinical pathways by 81.6% (relative risk (RR) 0.184 (95% CI: 0.072–0.471); *p* = 0.002) for Center A and by 88.6% (RR 0.114 (95% CI: 0.016–0.816); *p* = 0.0471) for Center B, as illustrated in Figure 1.

The interrupted time series model shows a 77% reduction (RR 0.230 (95% CI: 0.167–0.316); *p* < 0.001) in broad-spectrum antibiotic prescriptions rates after the intervention with rates increasing monthly by 3.5% (RR 1.035 (95% CI: 1.025–1.045); *p* < 0.001) in the post periods in Center C.

Amoxicillin dosage adjusted from 53.7 (IQR:7.0) to 51.6 mg/kg/die (IQR:3.8) in Center B (Table 1 and pair-wise comparison in Supplementary Materials, Figure S1) and the median days of therapy (DOT) met the recommended 10 days (8.0 vs. 10.0; *p* < 0.001) after clinical pathways implementation in Center A (Table 1 and pair-wise comparison in Supplementary Materials, Figure S2).

### 2.2. Acute Otitis Media—Total

Overall, 3980 AOM visits were assessed, and 3039 met the inclusion criteria. In Center A and C, a significant difference was reported in age class in the various periods (Supplementary Materials, Table S2).

After ASP implementation, “wait-and-see” approach rates were higher in Center A (from 21.6% to 34.1%; *p* = 0.006) and amoxicillin prescriptions rates increased in Center A and B, with a concomitant decrease in broad-spectrum antibiotic prescriptions rates (Center A: from 67.3% to 38.1%; *p* < 0.001, Center B: from 56.6% to 33.6%; *p* < 0.001), especially cephalosporins prescriptions (Table 2).

In Center C, the highest “wait-and-see” approach rate (32.7%) and the lowest broad-spectrum antibiotic prescriptions rate (68.3%) were reported in the semester following the ASP. Initially, the intervention doubled the “wait-and-see” approach rates (RR 2.510 (95% CI: 1.832–3.349); *p* < 0.001), even if rates did not remain stable, but decreased by 3.6% monthly (RR 0.036 (95% CI: 0.964–0.955); *p* < 0.001).

The broad-spectrum antibiotic prescriptions rates decreased by 29.5% (RR 0.705 (95% CI: 0.538–0.923); *p* = 0.011) in Center A and by 55.2% (RR 0.448 (95% CI: 0.235–0.856); *p* = 0.015) in Center B after the clinical pathways implementation. In Center C, the intervention reduced broad-spectrum antibiotic prescriptions by 41.1% (RR 0.589 (95% CI: 0.470–0.737); *p* < 0.001), but in the following semesters, the broad-spectrum antibiotic prescriptions rates increased by 1.5% monthly (RR 1.015 (95% CI 1.009–1.022); *p* < 0.001). The interrupted time series are shown in Figure 1.

Amoxicillin dosage increased from 50.0 (IQR:0.0) to 75.0 (IQR:5.0) mg/kg/die in Center A (*p* < 0.001), similarly to Center B (from 56.7 (IQR:14.3) to 75.0 (IQR:7.1) mg/kg/die). Co-amoxiclav dosage increase was also significant for both centers (Supplementary Materials, Figure S3).

**Figure 2:**
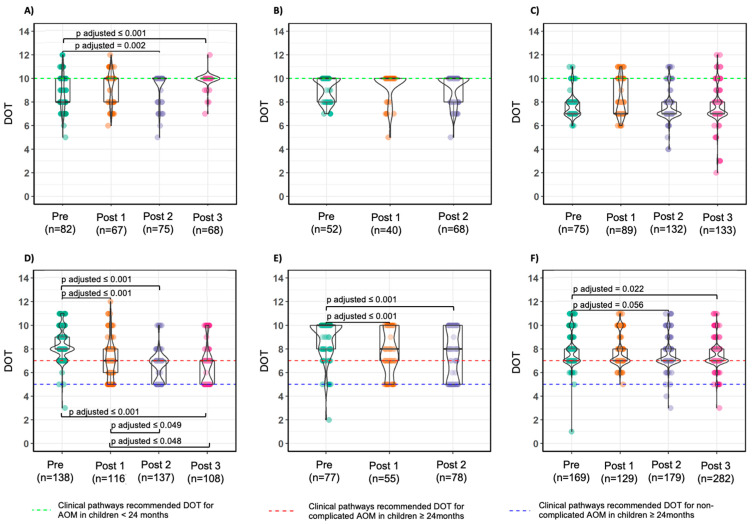
Distribution of days of therapy for non-complicated acute otitis media in the different periods in Center A (**A**,**D**), Center B (**B**,**E**), and Center C (**C**,**F**) stratified by age class (<24 months: **A**–**C**; ≥24 months: **D**–**F**) with pair-wise comparison. The dots represent the granular data, horizontal lines are median and IQR; whiskers extend to the minimum and maximum within 1.5 times the IQR. Violin plots present quantifications. The dotted green line represents the DOT recommended in the clinical pathways for acute otitis media in children <24 months, while the dotted blue line represents clinical pathways recommended DOT for non-complicated AOM in children ≥24 months and the dotted red line represents clinical pathways recommended DOT for complicated AOM in children ≥24 months.

DOT in children <24 months varied significantly from 8.0 (IQR:2.0) to 10.0 (IQR:0.0) just in Center A (Figure 2).

In older children, a variation was noted in Center A (from 8.0 (IQR:1.0) to 7.0 (IQR:2.0) DOT; *p* < 0.001) and in Center B (from 10.0 (IQR:2.0) to 8.0 (IQR:5.0) DOT; *p* < 0.001), while in Center C the median DOT remained 7.0 for the all periods in both age class.

### 2.3. Acute Otitis Media—Sensitivity Analysis

In 58.0% (2310/3980) of the AOM diagnoses, there was no sign of otorrhea reported.

In Center A and Center B, co-amoxiclav prescriptions decreased after clinical pathway introduction (Center A: from 43.6% to18.6%; *p* < 0.001, Center B: from 33.3% to 19.7%; *p* = 0.035), whereas a pattern similar to total AOM prescription was noted for Center C (Supplementary Materials, Table S3).

The “wait-and-see” approach rates increased by 68.8% after the intervention and stabilized in Center A (RR 1.688 (95% CI: 1.116–2.552); *p* = 0.013), while in Center C, the intervention was significant in increasing the “wait-and-see” approach rates immediately after the intervention (RR: 2.363 (95% CI: 1.774–3.148); *p* < 0.001); then, rates decreased monthly by 3.3% (RR: 0.967 (95% CI: 0.959–0.976); *p* < 0.001)).

The interrupted time series analysis (Figure 1) confirmed previous findings for Center A and Center C on broad-spectrum antibiotic prescriptions rates, but the reduction by 54.1% (RR 0.459 (95% CI: 0.198–1.060)) in Center B was non-significant (*p* = 0.068).

Amoxicillin and co-amoxiclav dosages did not differ from total AOM findings (Supplementary Materials, Figure S4), whereas median DOT for older children decreased significantly from 8.0 (IQR:2.5) to 5.0 (IQR:2.0) DOT (Supplementary Materials Figure S5) in Center A.

## 3. Discussion

In this multicentric study, clinical pathways combined with educational lectures proved to be a feasible ASP in decreasing and maintaining broad-spectrum antibiotic prescriptions rates over time for both AOM and pharyngitis (Center A and B). On the other hand, clinical pathways alone failed to maintain the low broad-spectrum prescription rates achieved after the intervention (Center C).

The ASPs reduced co-amoxiclav and cephalosporins prescription rates for pharyngitis by around 80% in all centers. In Center C, the reduction was not maintained after the first semester, possibly due to prescribers’ fear of coinfection in Group A Streptococcus carriers [11]. DOT changes reflected prescriptions variation in Center A and Center C, reaching the 10 DOT suggested for amoxicillin in order to decolonize the oropharynx in the Center A. It is unlikely that the recommended duration of amoxicillin is less than the clinical pathway indications, even if penicillin V administered four times daily for five days could represent an alternative regimen in older children with Group A Streptococcus pharyngitis, where commercially available [12,13].

“Wait-and-see” approach rates increased significantly in non-complicated AOM with stable rates after the ASPs implementation. The “wait-and-see” approach was introduced in the early 2000s and was included in most guidelines for the treatment of AOM [14,15] with variation in its applicability. The clinical pathways suggested a “wait-and-see” approach in children with non-severe AOM: if aged 6–24 months just with the unilateral form. In all cases, parents’ compliance and the possibility of a follow-up 48–72 h after was needed. The lack of rate variation in Center B could imply that emergency physicians did not feel that it was an appropriate strategy for their setting [16]. Similar to pharyngitis, ASPs were effective in broad-spectrum antibiotic prescriptions rate reduction for AOM. Nonetheless, in Center C, broad-spectrum antibiotic prescriptions rates for non-complicated AOM raised in the last semesters, increasing the possibility to cause more adverse events (i.e., vomiting, diarrhea, rash), in a country where only fixed 7:1 ratio packages are marketed, and the risk of selection of resistant bacteria in the community [17,18,19]. The same might be the reason why in Center B a more cautious behavior was noted for administering co-amoxiclav, especially at high dosage. Literature findings suggest that a higher co-amoxiclav ratio seems to be associated with fewer side effects without reducing clinical efficacy, but clinical pathways are based on the available medicine formulary to be easily adaptable.

In non-complicated AOM, five median DOT for older children was achieved in Center A’s final semester, reflecting prescribers’ initial discomfort with short-course treatment. Overall median DOT might not be the most suitable indicator in assessing ASP efficacy on treatment duration when higher rates of second-line therapy (i.e., cephalosporins) are observed, such as in Center C. A possible solution is calculating the median DOT stratified by different drugs.

Our study has several caveats: first, its retrospective nature and difficulties in assessing the reasons why broad-spectrum antibiotics were prescribed. However, the same study nature allowed us to exclude the Hawthorne effect that sometimes could be argued to play a major role in the ASP success. Second, no control groups were selected; hence, we cannot conclude with a high degree of certainty that the variation in broad-spectrum antibiotic prescriptions rates is caused by the ASP implementation without considering other possible explanations. On the other hand, no policy restricting antibiotic prescriptions was implemented in the different centers during the study period, nor were there any shortages in Group A Streptococcus-rapid tests. Third, patient follow-up was beyond the aim of the study and was, therefore, not performed. Although it is possible that patients receiving broad-spectrum antibiotic prescriptions had better clinical outcomes, preliminary results reported that there was no difference in treatment failure rate nor in adverse event rate in the first and second semesters [10]. Fourth, even if the first part of clinical pathways was focused on diagnosis, with a particular emphasis on signs to be considered, clinicians were not tested on the use of pneumatic otoscope nor Group A Streptococcus -rapid tests. Finally, it can be argued that differences in prescriptions may reflect variations in local bacteria resistance, but clinical pathways were developed with microbiologists from different centers, and clinicians were able to adapt them according to local microbiological data.

Despite clinical pathways proving to be a feasible ASP tool with rapid implementation and reduced applicability cost, this study revealed that without combining it with continuous education, it might have no lasting effect. In fact, the Infectious Disease Society of America recommends two core strategies to be implemented together, even if most pediatric ASPs consist of just one intervention. According to a recent systematic review, most of the studies do not report a long follow up, and few report negative results, though publication bias might contribute to this [6].

A study trial where an ASP based on continuous clinician-specific education combined with audit and feedback was implemented in the USA outpatients setting found that following the removal of the audit and feedback, the initial reduction in broad-spectrum antibiotic prescriptions to children with acute respiratory tract infection was lost [20]. The authors believed that audit and feedback was a vital element of the ASP and continuous, active efforts are required to sustain initial improvements in prescribing attitude. Moreover, another trial in a similar setting, comparing different ASPs, found out that only the peer-comparison approach maintained prescription rates lower than the control group after stopping the interventions [21].

Clinicians and researchers interested in implementing an ASP should carefully consider their options in order to avoid inefficiency. A possible improvement in the ASPs proposed lay on the analysis timing. Data were manually collected in condition-specific data collection forms, requiring ad hoc specialists to perform data entry. One solution could be conducting random day-point prevalence surveys every couple of weeks or months in setting with rapid patients turn-over, thus limiting the time dedicated to the collection and providing more rapid estimates on prescribing behaviors [22,23,24]. Secondly, having IT support to aid in developing real-time indicators will allow for rapid intervention and identification of root causes in cases of prescriber non-adherence to the ASPs [25]. Lastly, in our study, we assessed ASPs’ impact and sustainability for conditions with a higher incidence in the cold season, and for this reason, lectures were specifically held in the first months of the season; in the case of developing an ASP for a condition with no such seasonal variation (i.e., sepsis), the ASP team could opt for lectures closer together in time.

## 4. Materials and Methods

### 4.1. Study Design

This multicenter quasi-experimental study assesses the ASPs impact on antibiotics prescribing in the pediatric emergency department of three different hospitals: two tertiary-level university hospitals (Azienda Ospedale-Università, Padova and Ospedale Pediatrico Bambino Gesù, Rome, having around 24,000 and 56,000 yearly emergency room visits, respectively) and one secondary-level hospital (Ospedale Ca’ Foncello, Treviso, having around 14,000 yearly emergency room visits). Each institution was randomly named with a capital letter (Center A, B, C) to keep them intentionally anonymous.

**Figure 3:**
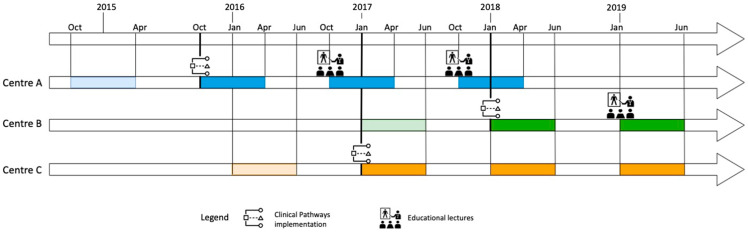
Study flow chart of the ASPs’ implementation in the three centers.

The different periods considered were: one semester before and three semesters after implementation for Center A and C (Center A: 15 October 2014–15 April 2015; 15 October 2015–15 April 2016; 15 October 2016–15 April 2017; 15 October 2017–15 April 2018; Center C: 1 January 2016–30 June 2016; 1 January 2017–30 June 2017; 1 January 2018–30 June 2018; 1 January 2019–30 June 2019) and one semester before and two semesters after implementation for Center B (1 January 2017–30 June 2017; 1 January 2018–30 June 2018; 1 January 2019–30 June 2019). The study flowchart is shown in Figure 3.

During the study period, in the pediatric emergency departments of the three hospitals, there were physicians that worked on a daily basis, specialist consultants that worked depending on the requests and residents that worked on a daily basis but changed every couple of months.

### 4.2. Intervention

Two different ASPs were adopted: for Center A and B, the intervention consisted of clinical pathway implementation and dissemination as laminated pocket-cards with yearly educational lectures for residents and pediatricians, and for Center C, the intervention consisted only of the implementation of clinical pathways with the possibility of consulting in the hospital intranet.

**Figure A1:**
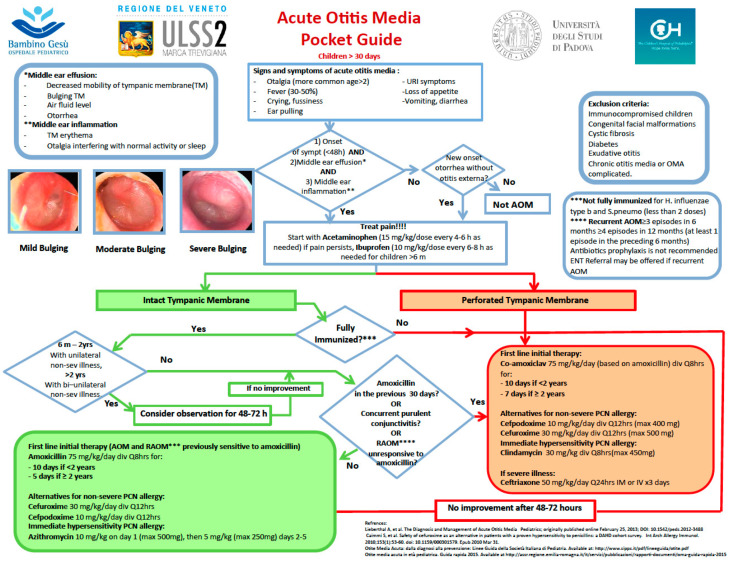
Clinical pathway for acute otitis media for children older than 30 days of age.

**Figure A2:**
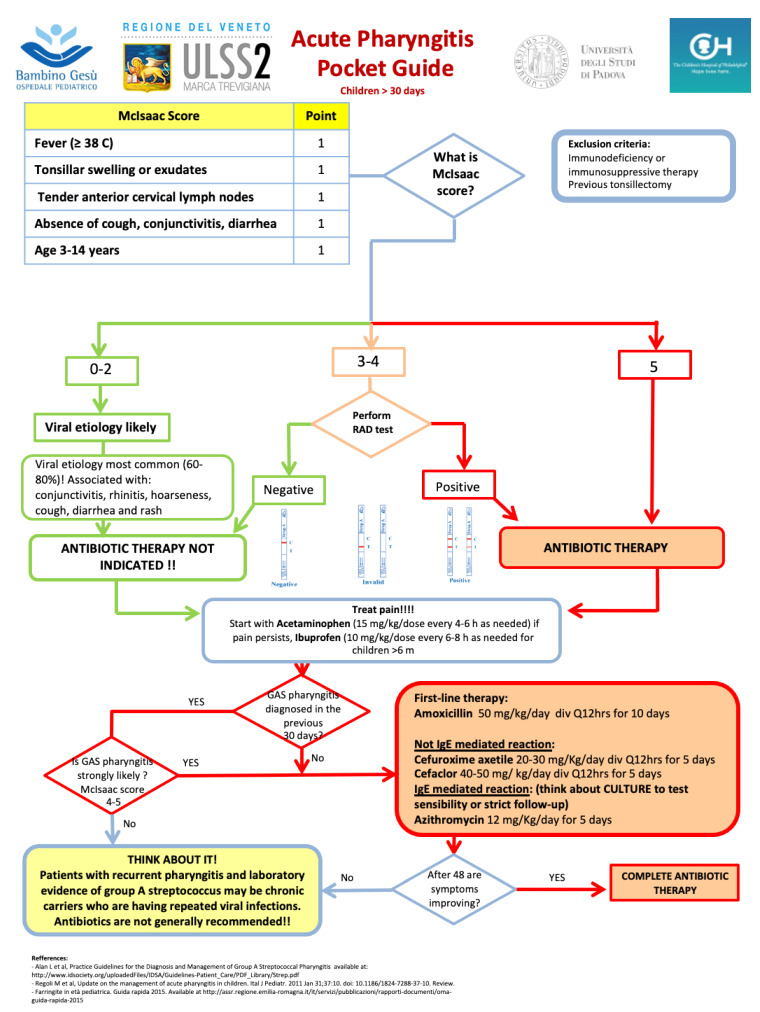
Clinical pathway for acute acute pharyngitis for children older than 30 days of age.

A multidisciplinary group of experts from each center, in collaboration with the Division of Pediatric Infectious Diseases of the Children’s Hospital of Philadelphia, developed the clinical pathways for pharyngitis and AOM that were adapted to the centers’ standard of care with no changes in the algorithm (Appendix A Figure A1 and Appendix B Figure A2).

The educational lectures addressed to residents, structured physicians and specialists and held by the center ASP team, consisted of two hours of training on the diagnosis and treatments of AOM and pharyngitis with a focus on the rational for antibiotic prescribing.

### 4.3. Population and Case Definition

All consecutive patients aged two months to 14 years with an International Classification of Diseases, 9th Revision, Clinical Modification code, or descriptive diagnosis of AOM or pharyngitis admitted to the pediatric emergency department in one of the three centers were included.

General exclusion criteria were immunodeficiency or immunosuppressive therapy, concomitant bacterial infections or systemic bacterial infection, craniofacial abnormalities, chronic diseases (i.e., diabetes, cystic fibrosis), and ongoing antibiotic therapy at admission. Pharyngitis exclusion criteria were previous tonsillectomy, periodic fever, aphthous stomatitis, pharyngitis and adenitis syndrome, and admission to the pediatric emergency department for feeding difficulties. AOM exclusion criteria were tympanostomy tubes at the time of diagnosis, chronic otitis media, and AOM complicated by mastoiditis.

Pediatric emergency department visits occurring for the same patient greater than 30 days apart were analyzed as separate events.

All AOM episodes with otorrhea were considered as complicated AOM; the remaining episodes were considered as non-complicated.

Broad-spectrum antibiotics were defined as β-lactam and β-lactamase inhibitor combinations, second- and third-generation cephalosporins, fluoroquinolones, and macrolides. Topic antibiotics (i.e., ciprofloxacin ear drops) were not considered.

### 4.4. Outcomes

The following aspects of antibiotic prescriptions for pharyngitis and AOM were assessed:

1. “Wait-and-see” approach rates (AOM only);
2. Broad-spectrum antibiotic prescriptions rates;
3. Rates by active agent;
4. Amoxicillin and co-amoxiclav dosage, expressed in mg/kg/day (Center A and Center B only);
5. Duration of therapy expressed in DOT.

The “wait-and-see” approach was defined as AOM episodes with no antibiotic prescription.

### 4.5. Data Collection and Sample Size Calculation

All clinical, demographic, diagnostic, and prescription data were manually collected from electronic medical records, using a password protected REDCap 10.0.1-© 2020 (Vanderbilt University) data collection form and stored on a secured server at the University of Padova. Privacy was guaranteed by assigning each patient a unique study-specific number and not collecting personally identifying data.

Assuming that before ASP implementation, (i) in 10% of AOM episodes a “wait-and-see” approach would be chosen and in 45% of pharyngitis episodes no antibiotic would be prescribed, (ii) the broad-spectrum antibiotic prescriptions rates would be 50%, (iii) broad-spectrum antibiotic prescriptions would decrease by 25%, (iv) 15% of the episodes did not fulfill the inclusion criteria, (v) a two-tailed Type I error of 0.05 is used, and (vi) the study is required to have at least a power of 70%, we estimated a minimum sample size of 330 pharyngitis and 260 AOM episodes per period per center to detect a significant decrease in broad-spectrum antibiotic prescriptions. The power for estimating the difference between independent proportions was calculated using G Power 3.1.9.4-© 1992–2019 (Universitat Kiel, Germany) [26].

The investigations were carried out following the rules of the Declaration of Helsinki of 1975 (https://www.wma.net/what-we-do/medical-ethics/declaration-of-helsinki/), revised in 2013. This study was approved by the Ethical Committees of all Centers (3737/AO/16). Due to the nature of the study (observational retrospective), no informed consent was required from the patients.

### 4.6. Data Analysis

Single center results in the different periods were summarized as numbers and percentages (categorical variables) and as median and interquartile range (continuous variables). Categorical variables were compared with χ2 or Fisher’s 2-tailed exact test in a contingency table *r* x *c*; a Fisher test was used when the value in any of the cells of the contingency table was below five. Continuous variables were compared with a non-parametric Kruskal-Wallis rank sum test; for pair-wise comparisons, we used Dwass-Steele-Critchlow-Fligner all-pairs test adjusted with Holm method. Since different DOT are recommended depending on child age, DOT analysis was stratified according to age class (2–23 months of age vs. 2–14 years of age).

An interrupted time series analysis supposing an abrupt step change in monthly significative outcomes (1 and 2) using quasi-Poisson regression models was used to determine the effect of the intervention. [27]“Wait-and-see” approach, log-transformed total AOM episodes, a variable representing the frequency in months in which observations were taken, and a dummy variable indicating the pre- and post-intervention periods were considered. For outcome (2), broad-spectrum antibiotic prescriptions and log-transformed total antibiotic prescriptions were considered together with a frequency variable and a dummy variable previously specified. A seasonal adjustment was not necessary since the same calendar months were considered to control for effects. Autocorrelation was assessed, examining the plot for residuals and the partial autocorrelation function. The corresponding relative risk and 95% confidence interval (95% CI) according to normal approximation were calculated.

Outcome data were sometimes missing (0–20%, Supplementary Materials, Table S1). If variable data were missing completely at random [28] and restricting the analysis would not have resulted in a significant loss of information or biased estimation, listwise deletion was performed (i.e., dosage); in the opposite case (i.e., DOT), group-wise predictive mean matching within the fully conditional specification algorithm was used to fit the missing data [29].

A sensitivity analysis was conducted for non-complicated AOM episodes. Data were analyzed using R statistical software (version 3.6.3, Vienna, Austria) for Windows [30]. The multiple imputation was performed with the “mice” and “miceadds” packages [31]. Figures were created with the packages “ggplot2” [32] and “ggstatsplot” [33]. For brevity, statistical parameters were included in figures displaying pair-wise comparisons. Statistical significance was set at the 0.05 level and *p* values were two-sided.

## 5. Conclusions

To the best of our knowledge, this is the first attempt to study the efficacy and sustainability of ASPs over time based on clinical pathways in pediatric emergency departments. Our findings suggest that clinical pathways paired with continuous education can be effective in reducing broad-spectrum antibiotic prescription and in reaching target treatment duration. Researchers should push for efficient assessment and publication of intervention sustainability in order to help other clinicians in choosing the most suitable ASP for their setting.

## Figures and Tables

**Table 1:** Treatment option for pharyngitis in the different periods in the three Centers.

	Center A					Center B				Center C				
Period	Pre	Post 1	Post 2	Post 3	*p*-Value	Pre	Post 1	Post 2	*p*-Value	Pre	Post 1	Post 2	Post 3	*p*-Value
	298	364	326	264		290	241	251		363	217	316	319	
No antibiotic, N (%)	147 (49.3)	198 (54.4)	120 (36.8)	129 (48.9)	<0.001	135 (46.6)	140 (58.1)	122 (48.6)	0.021	23 (6.3)	15 (6.9)	44 (13.9)	23 (7.2)	0.002
Antibiotic therapy, N (%)	151 (50.7)	166 (45.6)	206 (63.2)	135 (51.1)		155 (53.4)	101 (41.9)	129 (51.4)		340 (93.7)	202 (93.1)	272 (86.1)	296 (92.8)	
Amoxicillin, N (%)	81 (53.6)	155 (93.4)	192 (93.2)	133 (98.5)	<0.001	108 (69.7)	97 (96.0)	114 (88.4)	<0.001	42 (12.4)	144 (71.3)	133 (48.9)	84 (28.4)	<0.001
Broad spectrum, N (%)	70 (46.4)	11 (6.6)	14 (6.8)	2 (1.5)		47 (30.3)	4 (4.0)	15 (11.6)		298 (87.6)	58 (28.7)	139 (51.1)	212 (71.6)	
Co-amoxiclav, N (%)	60 (39.7)	5 (3.0)	9 (4.4)	1 (0.7)	<0.001	28 (18.1)	1 (1.0)	5 (3.9)	<0.001	249 (73.2)	45 (22.3)	102 (37.5)	170 (57.4)	<0.001
Cephalosporins, N (%)	10 (6.6)	6 (3.6)	4 (1.9)	1 (0.7)	0.029	14 (9.0)	3 (3.0)	7 (5.4)	0.131	17 (5.0)	4 (2.0)	25 (9.2)	33 (11.1)	<0.001
Macrolides, N (%)	0 (0.0)	0 (0.0)	1 (0.5)	0 (0.0)		5 (3.2)	0 (0.0)	3 (2.3)	0.203	32 (9.4)	9 (4.5)	12 (4.4)	9 (3.0)	0.004
Amoxicillin dosage, Median [IQR], mg/kg/die	50.0 [0.0]	50.0 [0.0]	50.0 [1.0]	50.0 [0.0]	0.288	53.7 [7.0]	50.6 [3.5]	51.6 [3.8]	<0.001	/	/	/	/	/
Days of therapy, Median [IQR]	8.0 [3.0]	10.0 [0.0]	10.0 [0.0]	10.0 [0.0]	<0.001	10.0 [1.0]	10.0 [0.0]	10.0 [0.0]	0.622	7.0 [0.0]	7.0 [3.0]	8.00 [1.0]	7.0 [1.0]	<0.001

IQR = Interquartile range.

**Table 2:** Treatment option for acute otitis media in the different periods in the three centers.

	Center A					Center B				Center C				
Period	Pre	Post 1	Post 2	Post 3	*p*-Value	Pre	Post 1	Post 2	*p*-Value	Pre	Post 1	Post 2	Post 3	*p*-Value
	281	273	299	267		139	105	151		302	324	387	481	
Wait and see, N (%)	61 (21.7)	90 (33.0)	87 (29.1)	91 (34.1)	0.006	10 (7.2)	10 (9.5)	5 (3.3)	0.116	58 (19.2)	106 (32.7)	76 (19.6)	66 (13.7)	<0.001
Antibiotic treatment, N (%)	220 (78.3)	183 (67.0)	212 (70.9)	176 (65.9)		129 (92.8)	95 (90.5)	146 (96.7)		244 (80.8)	218 (67.3)	311 (80.4)	415 (86.3)	
Amoxicillin, N (%)	72 (32.7)	95 (51.9)	113 (53.3)	109 (61.9)	<0.001	56 (43.4)	66 (69.5)	97 (66.4)	<0.001	30 (12.3)	69 (31.7)	59 (19.0)	73 (17.6)	<0.001
Broad spectrum, N (%)	148 (67.3)	88 (48.1)	99 (46.7)	67 (38.1)		73 (56.6)	29 (30.5)	49 (33.6)		214 (87.7)	149 (68.3)	252 (81.0)	342 (82.4)	
Co-amoxiclav, N (%)	100 (45.5)	68 (37.2)	83 (39.2)	62 (35.2)	0.169	44 (34.1)	22 (23.2)	34 (23.3)	0.081	165 (67.6)	121 (55.5)	184 (59.2)	293 (70.6)	<0.001
Cephalosporins, N (%)	44 (20.0)	16 (8.7)	14 (6.6)	5 (2.8)	<0.001	28 (21.7)	5 (5.3)	15 (10.3)	<0.001	43 (17.6)	26 (11.9)	62 (19.9)	45 (10.8)	0.002
Macrolides, N (%)	4 (1.8)	4 (2.2)	1 (0.5)	0 (0.0)	0.119	1 (0.8)	2 (2.1)	0 (0.0)	0.187	6 (2.5)	2 (0.9)	6 (1.9)	4 (1.0)	0.364
Fluoroquinolones, N (%)	0 (0.0)	0 (0.0)	1 (0.5)	0 (0.0)		0 (0.0)	0 (0.0)	0 (0.0)		0 (0.0)	0 (0.0)	0 (0.0)	0 (0.0)	
Amoxicillin dosage, Median [IQR], mg/kg/die	50.0 [0.0]	75.0 [25.0]	75.0 [4.0]	75.0 [0.0]	<0.001	57.6 [14.3]	73.0 [8.6]	75.0 [7.1]	<0.001	/	/	/	/	
Co-amoxiclav dosage, Median [IQR], mg/kg/die	50.0 [10.0]	75.0 [25.0]	73.0 [15.2]	75.0 [5.0]	<0.001	56.7 [7.9]	63.1 [23.9]	68.6 [15.2]	<0.001	/	/	/	/	
Days of therapy, Median [IQR], 2–23 months	8.0 [2.0]	10.0 [2.0]	10.0 [0.0]	10.0 [0.0]	<0.001	10.0 [2.0]	10.0 [0.0]	10.0 [2.0]	0.170	7.0 [1.0]	7.0 [3.0]	7.0 [1.0]	7.0 [1.0]	0.024
Days of therapy, Median [IQR], ≥24 months	8.0 [1.0]	7.0 [2.0]	7.0 [2.0]	7.0 [2.0]	<0.001	10.0 [2.0]	8.0 [3.0]	8.0 [5.0]	<0.001	7.0 [1.0]	7.0 [1.0]	7.0 [1.0]	7.0 [1.0]	<0.001

IQR = Interquartile range.

## Supplementary Materials

The following are available online at https://www.mdpi.com/2079-6382/9/12/867/s1, Table S1. Missing data in the different periods in the three centers (A, B, C); Table S2. Demographic characteristics of the included and excluded patients with pharyngitis and AOM in the different periods in the three centers (A, B, C); Table S3. Treatment option for non-complicated AOM in the different periods in the three centers (A, B, C); Figure S1. Distribution of amoxicillin dosage for pharyngitis among different periods in Center A and B with pairwise comparison; Figure S2. Distribution of days of therapy for pharyngitis among different periods in the three centers (A, B, C) with pairwise comparison; Figure S3. Distribution of co-amoxiclav (A, B) and amoxicillin (C, D) dosage for acute otitis media among different periods in Center A (A, C) and Center B (B, D) with pairwise comparison; Figure S4. Distribution of co-amoxiclav (A, B) and amoxicillin (C, D) dosage for non-complicated acute otitis media among different periods in Center A (A, C) and Center B (B, D) with pairwise comparison; Figure S5. Distribution of days of therapy (DOT) for acute otitis media in the different periods in Centre A (A, D), Centre B (B, E) and Centre C (C, F) stratified by age class (<24 months: A, B, C; ≥24 months: D, E, F) with pair wise comparison.

## References

[1] Sturkenboom M.C.J.M., Verhamme K.M.C., Nicolosi A., Murray M.L., Neubert A., Caudri D., Picelli G., Sen E.F., Giaquinto C., Cantarutti L. (2008). Drug use in children: Cohort study in three European countries. BMJ.

[2] Messina F., Clavenna A., Cartabia M., Piovani D., Bortolotti A., Fortino I., Merlino L., Bonati M. (2019). Antibiotic prescription in the outpatient paediatric population attending emergency departments in Lombardy, Italy: A retrospective database review. BMJ Paediatr. Open.

[3] Barbieri E., Donà D., Cantarutti A., Lundin R., Scamarcia A., Corrao G., Giaquinto C. (2019). Antibiotic prescriptions in acute otitis media and pharyngitis in Italian pediatric outpatients. Italy J. Pediatr..

[4] Marchisio P., Esposito S., Picca M., Baggi E., Terranova L., Orenti A., Biganzoli E., Principi N., Gallia P., Mazzucchi E. (2017). Prospective evaluation of the aetiology of acute otitis media with spontaneous tympanic membrane perforation. Clin. Microbiol. Infect..

[5] Kronman M.P., Zhou C., Mangione-Smith R. (2014). Bacterial prevalence and antimicrobial prescribing trends for acute respiratory tract infections. Pediatrics.

[6] Donà D., Barbieri E., Daverio M., Lundin R., Giaquinto C., Zaoutis T., Sharland M. (2020). Implementation and impact of pediatric antimicrobial stewardship programs: A systematic scoping review. Antimicrob Resist. Infect Control.

[7] Samore M.H., Bateman K., Alder S.C., Hannah E., Donnelly S., Stoddard G.J., Haddadin B., Rubin M.A., Williamson J., Stults B. (2005). Clinical decision support and appropriateness of antimicrobial prescribing: A randomized trial. JAMA.

[8] Donà D., Zingarella S., Gastaldi A., Lundin R., Perilongo G., Frigo A.C., Hamdy R.F., Zaoutis T., Da Dalt L., Giaquinto C. (2018). Effects of clinical pathway implementation on antibiotic prescriptions for pediatric community-acquired pneumonia. PLoS ONE.

[9] Donà D., Luise D., Barbieri E., Masiero N., Maita S., Antoniello L., Zaoutis T., Giaquinto C., Gamba P. (2020). Effectiveness and Sustainability of an Antimicrobial Stewardship Program for Perioperative Prophylaxis in Pediatric Surgery. Pathogens.

[10] Dona D., Baraldi M., Brigadoi G., Lundin R., Perilongo G., Hamdy R.F., Zaoutis T., Da Dalt L., Giaquinto C. (2018). The Impact of Clinical Pathways on Antibiotic Prescribing for Acute Otitis Media and Pharyngitis in the Emergency Department. Pediatr. Infect Dis. J..

[11] Spuesens E.B.M., Fraaij P.L.A., Visser E.G., Hoogenboezem T., Hop W.C.J., Van Adrichem L.N.A., Weber F., Moll H.A., Broekman B., Berger M.Y. (2013). Carriage of Mycoplasma pneumoniae in the upper respiratory tract of symptomatic and asymptomatic children: An observational study. PLoS Med..

[12] Ståhlgren G.S., Tyrstrup M., Edlund C., Giske C.G., Mölstad S., Norman C., Rystedt K., Sundvall P.-D., Hedin K. (2019). Penicillin V four times daily for five days versus three times daily for 10 days in patients with pharyngotonsillitis caused by group A streptococci: Randomised controlled, open label, non-inferiority study. BMJ.

[13] Pichichero M.E., Post T.W. (2015). Treatment and prevention of streptococcal pharyngitis. Up to Date.

[14] Lieberthal A.S., Carroll A.E., Chonmaitree T., Ganiats T.G., Hoberman A., Jackson M.A., Joffe M.D., Miller D.T., Rosenfeld R.M., Sevilla X.D. (2013). The diagnosis and management of acute otitis media. Pediatrics.

[15] SIP Guidelines. https://sip.it/2019/06/17/gestione-otite-media-acuta-linee-guida/.

[16] Fischer T., Singer A.J., Lee C., Thode H.C. (2007). National trends in emergency department antibiotic prescribing for children with acute otitis media, 1996–2005. Acad. Emerg. Med..

[17] Arguedas A., Dagan R., Leibovitz E., Hoberman A., Pichichero M., Paris M. (2006). A multicenter, open label, double tympanocentesis study of high dose cefdinir in children with acute otitis media at high risk of persistent or recurrent infection. Pediatr. Infect Dis. J..

[18] Agenzia Italiana del Farmaco—Italian Medicine Agency. https://farmaci.agenziafarmaco.gov.it/bancadatifarmaci/cerca-per-principioattivo?princ_att=Amoxicillina%20e%20inibitori%20enzimatici.

[19] Hoberman A., Paradise J.L., Rockette H.E., Jeong J.-H., Kearney D.H., Bhatnagar S., Shope T.R., Muñiz G., Martin J.M., Kurs-Lasky M. (2017). Reduced-Concentration Clavulanate for Young Children with Acute Otitis Media. Antimicrob. Agents Chemother..

[20] Gerber J.S., Prasad P.A., Fiks A.G., Localio A.R., Bell L.M., Keren R., Zaoutis T.E. (2014). Durability of benefits of an outpatient antimicrobial stewardship intervention after discontinuation of audit and feedback. JAMA.

[21] Linder J.A., Meeker D., Fox C.R., Friedberg M.W., Persell S.D., Goldstein N.J., Doctor J.N. (2017). Effects of Behavioral Interventions on Inappropriate Antibiotic Prescribing in Primary Care 12 Months After Stopping Interventions. JAMA.

[22] Velasco-Arnaiz E., Simó-Nebot S., Ríos-Barnés M., Ramos M.G.L., Monsonís M., Urrea-Ayala M., Jordan I., Mas-Comas A., Casadevall-Llandrich R., Ormazábal-Kirchner D. (2020). Benefits of a Pediatric Antimicrobial Stewardship Program in Antimicrobial Use and Quality of Prescriptions in a Referral Children’s Hospital. J. Pediatr..

[23] De Luca M., Donà D., Montagnani C., Vecchio A.L., Romanengo M., Tagliabue C., Centenari C., D’Argenio P., Lundin R., Giaquinto C. (2016). Antibiotic Prescriptions and Prophylaxis in Italian Children. Is It Time to Change? Data from the ARPEC Project. PLoS ONE.

[24] Hsia Y., Lee B.R., Versporten A., Yang Y., Bielicki J., Jackson C., Newland J., Goossens H., Magrini N., Sharland M. (2019). Use of the WHO Access, Watch, and Reserve classification to define patterns of hospital antibiotic use (AWaRe): An analysis of paediatric survey data from 56 countries. Lancet Glob. Health.

[25] Bremmer D.N., Trienski T.L., Walsh T.L., Moffa M.A. (2018). Role of Technology in Antimicrobial Stewardship. Med. Clin. N. Am..

[26] Faul F., Erdfelder E., Buchner A., Lang A.-G. (2009). Statistical power analyses using G*Power 3.1: Tests for correlation and regression analyses. Behav. Res. Methods.

[27] Bernal J.L., Cummins S., Gasparrini A. (2017). Interrupted time series regression for the evaluation of public health interventions: A tutorial. Int. J. Epidemiol..

[28] Kang H. (2013). The prevention and handling of the missing data. Korean J. Anesthesiol..

[29] Kleinke K. (2018). Multiple imputation by predictive mean matching when sample size is small. Methodol. Eur. J. Res. Methods Behav. Soc. Sci..

[30] R Core Team (2019). R: A Language and Environment for Statistical Computing.

[31] Van Buuren S., Groothuis-Oudshoorn K. (2011). Mice: Multivariate Imputation by Chained Equations in R. J. Stat. Softw..

[32] Wickham H. (2016). Ggplot2: Elegant Graphics for Data Analysis.

[33] Patil I. (2018). Ggstatsplot: “ggplot2” Based Plots with Statistical Details. CRAN.

