# Supplementary material for: Impact and Sustainability of Antibiotic Stewardship in Pediatric Emergency Departments: Why Persistence Is the Key to Success
Source: Antibiotics (Basel). 2020 Dec 4;9(12):867. doi: 10.3390/antibiotics9120867 (PMC7761799; doi:10.3390/antibiotics9120867)
Supplement: Supplementary file 1 [file antibiotics-09-00867-s001.pdf]

Supplementary material

Article

Impact and Sustainability of Antibiotic Stewardship in Pediatric Emergency Departments: Why Persistence Is the Key to Success

Elisa Barbieri [1,*], Maia De Luca [2], Marta Minute [3], Carmen D'Amore [4], Marta Luisa Ciofi Degli Atti [4], Stefano Martelossi [3], Carlo Giaquinto [1], Liviana Da Dalt [5], Theoklis Zaoutis [6] and Daniele Dona [1]

[1] Division of Pediatric Infectious Diseases, Department of Women's and Children's Health, University of Padova, 35131 Padova, Italy; carlo.giaquinto@unipd.it (C.G.); daniele.dona@unipd.it (D.D.)

[2] Unit of Immune and Infectious Diseases, Academic Department of Pediatrics, Bambino Gesù Children's Hospital, IRCCS, 00165 Rome, Italy; maiadeluca@opbg.net

[3] Pediatric Unit, Ca' Foncello's Hospital, 31100 Treviso, Italy; marta.minute@aulss2.veneto.it (M.M.); stefano.martelossi@aulss2.veneto.it (S.M.)

[4] Unit of Clinical Epidemiology, Bambino Gesù Children's Hospital, IRCCS, 00165 Rome, Italy; carmen.damore@opbg.net (C.D.); marta.ciofidegliatti@opbg.net (M.L.C.D.A.)

[5] Pediatric Emergency Department, Department of Women's and Children's Health, University Hospital of Padua, 2-35128 Padova, Italy; liviana.dadalt@unipd.it

[6] Division of Infectious Diseases and the Center for Pediatric Clinical Effectiveness, Children's Hospital of Philadelphia, Philadelphia, PA 19104, USA; zaoutis@email.chop.edu

* Correspondence: elisa.barbieri.5@phd.unipd.it

Tables

1. Table S1 Missing data in the different periods in the three Centers (A, B, C).
2. Table S2 Demographic characteristics of included and excluded patients with pharyngitis and AOM in the different periods in the three Centers (A, B, C).
3. Table S3 Treatment option for non-complicated AOM in the different periods in the three Centers (A, B, C).

Figures

1. Figure S1 Distribution of amoxicillin dosage for pharyngitis among different periods in Centre A and B with pairwise comparison.
2. Figure S2 Distribution of days of therapy for pharyngitis among different periods in the three Centers (A, B, C) with pairwise comparison.
3. Figure S3 Distribution of co-amoxiclav (A, B) and amoxicillin (C, D) dosage for acute otitis media among different periods in Centre A (A, C) and Centre B (B, D) with pairwise comparison.
4. Figure S4 Distribution of co-amoxiclav (A, B) and amoxicillin (C, D) dosage for non-complicated acute otitis media among different periods in Centre A (A, C) and Centre B (B, D) with pairwise comparison.
5. Figure S5 Distribution of days of therapy (DOT) for acute otitis media in the different periods in Centre A (A, D), Centre B (B, E) and Centre C (C, F) stratified by age class (< 24 months: A, B, C; ≥ 24 months: D, E, F) with pair wise comparison.

Tables

Table S1. Missing data in the different periods in the three Centers (A, B, C).

Outcome	Centre A				Centre B			Centre C			
	Pre	Post 1	Post 2	Post 3	Pre	Post 1	Post 2	Pre	Post 1	Post 2	Post 3
**GAS pharyngitis episode**											
Amoxicillin dosage, N(%)	/	/	/	/	2/108 (1·9)	2/97 (2·1)	4/114 (3·5)				
DOT, N (%)	/	/	/	/	29/155 (18·7)	/	4/129 (3·1)	3/340 (0·9)	4/202 (2·0)	1/272 (0·4)	4/296 (1·4)
**AOM episodes**											
Amoxicillin dosage, N(%)	/	/	/	2/109 (1·8)	2/56 (4)	6/66 (9)	4/97 (4)	/	/	/	/
Co-amoxiclav dosage, N(%)	/	/	/	1/62 (1·6)	4/44 (9)	1/22 (4)	1/34 (3)	/	/	/	/
DOT, N (%)											
2-23 months	/	/	/	1/68 (1·5)	9/52 (17·3)	/	1/68 (1·5)	3/75 (4·0)	1/89 (1·1)	/	2/133 (1·5)
2-14 years	/	/	/	3/108 (2·8)	11/77 (14·3)	/	/	7/169 (4·1)	3/129 (2·3)	/	5/282 (1·8)
**Non-complicated AOM episodes**											
Amoxicillin dosage, N(%)	/	/	/	3/78 (3·8)	2/56 (3·6)	6/60 (10·0)	4/93 (4·3)	/	/	/	/
Co-amoxiclav dosage, N(%)	/	/	/	/	4/41 (9·8)	1/19 (5·3)	1/26 (3·8)	/	/	/	/
DOT, N (%)											
2-23 months	/	/	/	1/49 (2·0)	9/50 (18·0)	/	1/65 (1·5)	2/44 (4·5)	1/64 (1·6)	/	1/91 (1·1)
2-14 years	/	/	/	2/80 (2·5)	11/73 (15·1)	/	/	6/103 (5·8)	3/78 (3·8)	/	3/194 (1·5)

Table S2 Demographic characteristics of included and excluded patients with pharyngitis and AOM in the different periods in the three Centers (A, B, C).

	**Pre**		**Post 1**		**Post 2**		**Post 3**		**p value** [a]
	**I**	**E**	**I**	**E**	**I**	**E**	**I**	**E**	
**Centre A**									
Pharyngitis episodes, N	281	49	273	64	299	68	267	92	
Sex, Male, N(%)	165 (58·7)	27 (55·1)	154 (56·4)	35 (54·7)	156 (52·2)	42 (61·8)	150 (56·2)	52 (56·5)	0·454
Age class, 2-23 months, N (%)	96 (34·2)	15 (30·6)	78 (28·6)	13 (20·3)	86 (28·8)	32 (47·1)	77 (28·8)	22 (23·9)	0·397
Presence of otorrhea, N (%)	44 (15·7)		60 (22·0)		69 (23·1)		49 (18·4)		0·101
Ongoing antibiotic therapy, N (%)		24 (49·0)		17 (26·6)		34 (50·0)		29 (31·5)	
Exclusion criteria									
Chemotherapy, wound care or attend a hemodialysis clinic		/		2 (4·55)		/		1 (1·28)	
Chronic otitis media		11 (22·4)		20 (31·3)		6 (8·8)		46 (50·0)	
Concomitant diseases		/		1 (1·6)		17 (25·0)		9 (9·8)	
Craniofacial abnormalities		/		13 (20·3)		9 (13·3)		9 (9·8)	
Diabetes		/		1 (1·6)		/		/	
Essudative otitis		3 (6·1)		1 (1·6)		9 (13·3)		2 (2·2)	
Immunodeficiency or immunosuppressive therapy		/		2 (3·5)		4 (5·9)		1 (1·1)	
Patient hospitalized		/		/		2 (2·9)		1 (1·1)	
Systemic antibiotic therapy, intravenous		5 (10·2)		1 (1·6)		2 (2·9)		3 (3·3)	
Tympanostomy tubes at the time of diagnosis		/		1 (1·6)		1 (1·5)		1 (1·1)	
Acute otitis media episodes, N	298	89	364	80	326	90	264	49	
Sex, Male, N(%)	168 (56·4)	57 (64·0)	212 (58·2)	41 (51·2)	172 (52·8)	54 (60·0)	134 (50·8)	30 (61·2)	0·228
Age class, 2-35 months, N (%)	100 (33·6)	29 (32·6)	135 (37·1)	29 (36·2)	83 (25·5)	32 (35·6)	73 (27·7)	14 (28·6)	0·004
Ongoing antibiotic therapy, N (%)		52 (58·4)		40 (50·0)		51 (56·7)		35 (71·4)	
Exclusion criteria									
Chronic diseases		3 (3·4)		4 (5·0)		1 (1·1)		2 (4·1)	
Complicated disease		1 (1·1)						5 (10·2)	
Concomitant diseases		23 (25·8)		23 (28·8)		21 (23·3)		5 (10·2)	
Immunodeficiency or immunosuppressive therapy		1 (1·1)						2 (4·1)	
Patient hospitalized						11 (12·2)		4 (8·2)	
PFAPA[b]		1 (1·1)		2 (2·5)		3 (3·3)		1 (2·0)	
Previous tonsillectomy		2 (2·2)		3 (3·8)		2 (2·2)		1 (2·0)	
Systemic antibiotic therapy, intravenous									
**Centre B**									
Pharyngitis episodes, N	139	28	105	11	151	15			

Sex, Male, N(%)	71 (51·1)	14 (50·0)	62 (59·0)	7 (63·6)	75 (49·7)	10 (66·7)			0·662
Age class, 2-23 months, N (%)	56 (40·3)	9 (32·1)	40 (38·1)	2 (18·2)	68 (45·0)	4 (26·7)			0·027
Presence of otorrhea, N (%)	7 (5·0)		11 (10·5)		14 (9·3)				0·243
Ongoing antibiotic therapy, N (%)		25 (89·3)		9 (81·8)		13 (86·7)			
Exclusion criteria									
Chemotherapy, wound care or attend a hemodialysis clinic		/		/		/			
Chronic otitis media		/		2 (18·2)		/			
Concomitant diseases		1 (3·6)		/		/			
Craniofacial abnormalities		2 (7·1)		/		/			
Diabetes		/		/		/			
Essudative otitis		/		1 (9·1)		1 (6·7)			
Immunodeficiency or immunosuppressive therapy		/		/		/			
Patient hospitalized		1 (3·6)		/		/			
Systemic antibiotic therapy, intravenous		1 (3·6)		/		1 (6·7)			
Tympanostomy tubes at the time of diagnosis		/		/		/			
Acute otitis media episodes, N	290	65	241	31	251	43			
Sex, Male, N(%)	168 (57·9)	37 (56·9)	131 (54·4)	19 (61·3)	145 (57·8)	24 (55·8)			0·659
Age class, 2-35 months, N (%)	124 (42·8)	18 (27·7)	92 (38·2)	7 (22·6)	92 (36·7)	7 (16·3)			0·314
Ongoing antibiotic therapy, N (%)		60 (92·3)		24 (77·4)		38 (88·4)			
Exclusion criteria									
Chronic diseasees		2 (3·1)							
Complicated disease		3 (4·6)		1 (3·2)					
Concomitant diseases				3 (9·7)		3 (7·0)			
Immunodeficiency or immunosuppressive therapy		2 (3·1)		2 (6·5)					
Patient hospitalized									
PFAPA[b]									
Previous tonsillectomy						1 (2·3)			
Systemic antibiotic therapy, intravenous						1 (2·3)			
**Centre C**									
Pharyngitis episodes, N	302	159	324	163	387	173	481	149	
Sex, Male, N(%)	156 (51·7)[c]	83 (52·2)	177[c] (54·6)	75 (46·0)	212 (54·8)	95 (54·9)	269 (55·9)[c]	72 (48·3)	0·705
Age class, 2-23 months, N (%)	85 (28·1)	51 (32·1)	109 (33·6)	59 (36·2)	146 (37·7)	50 (28·9)	146 (30·4)	49 (32·9)	0·035
Presence of otorrhea, N (%)	108 (35·8)		87 (26·9)		116 (30·0)		134 (27·9)		0·068
Ongoing antibiotic therapy, N (%)		67 (42·1)		67 (41·1)		85 (49·1)		58 (38·9)	
Exclusion criteria									
Chemotherapy, wound care or attend a haemodialysis clinic		/		/		1 (0·69)		/	
Chronic otitis media		1 (0·6)		3 (1·8)		5 (2·9)		3 (2·0)	

Concomitant diseases		36 (22·6)		26 (16·0)		71 (41·0)		26 (17·4)	
Craniofacial abnormalities		13 (8·2)		1 (0·6)		1 (0·69)		3 (2·0)	
Diabetes		/		/		/		/	
Essudative otitis		3 (1·9)		8 (4·9)		6 (3·5)		3 (2·0)	
Immunodeficiency or immunosuppressive therapy		5 (3·1)		3 (1·8)		1 (0·6)		2 (1·3)	
Patient hospitalized		/		/		/		/	
Systemic antibiotic therapy, intravenous		89 (56·0)		97 (59·5)		15 10·34)		19 (20·88)	
Tympanostomy tubes at the time of diagnosis		9 (5·7)		8 (4·9)		3 (1·7)		3 (2·0)	
Acute otitis media episodes, N	363	248	217	208	316	164	319	218	
Sex, Male, N(%)	199 (54·8)	154 (62·1)	113 (52·1)	120 (57·7)	193 (61·1)	104 (63·4)	168 (52·7)	117 (53·7)	0·118
Age class, 2-35 months, N (%)	195 (53·7)	112 (45·2)	105 (48·4)	87 (41·8)	149 (47·2)	63 (38·4)	124 (38·9)	80 (36·7)	0·002
Ongoing antibiotic therapy, N (%)		239 (96·4)		139 (66·8)		159 (97·0)		167 (76·6)	
Exclusion criteria									
Chronic diseases		1 (0·4)		7 (3·4)				5 (2·3)	
Complicated disease				12 (5·8)				2 (0·9)	
Concomitant diseases		5 (2·0)		8 (3·8)		1 (0·6)		14 (6·4)	
Immunodeficiency or immunosuppressive therapy		2 (0·8)				1 (0·6)		2 (0·9)	
Patient hospitalized		1 (0·4)				3 (1·8)		6 (2·8)	
PFAPA[b]				2 (1·0)				7 (9·6)	
Previous tonsillectomy		34 (13·7)		36 (17·3)		43 (26·2)		27 (12·3)	
Systemic antibiotic therapy, intravenous									

I=Included cases, E= Excluded cases.

[a] included cases only, [b] Periodic fever, aphthous stomatitis, pharyngitis and adenitis syndrome , [c] Missing data = 1/302 (0·3%), 4/324 (1·2%), 1/481 (0·2%).

Table S3. Treatment option for non-complicated AOM in the different periods in the three Centers (A, B, C).

	**Centre A**					**Centre B**				**Centre C**				
	**Pre**	**Post 1**	**Post 2**	**Post 3**	**p value**	**Pre**	**Post 1**	**Post 2**	**p value**	**Pre**	**Post 1**	**Post 2**	**Post 3**	**p value**
N. of episodes	236	213	230	218		132	94	137		194	237	271	347	
Wait and see, N (%)	58 (24·5)	89 (41·8)	83 (36·1)	89 (40·8)	<0·001	9 (6·8)	10 (10·6)	5 (3·6)	0·109	47 (24·2)	95 (40·1)	66 (24·4)	62 (17·9)	<0·001
Antibiotic treatment, N (%)	179 (75·5)	124 (58·2)	147 (63·9)	129 (59·2)		123 (93·2)	84 (89·4)	132 (96·4)		147 (75·8)	142 (59·9)	205 (75·6)	285 (82·1)	

Amoxicillin, N (%)	68 (38·0)	80 (64·5)	106 (72·1)	101 (78·3)	<0·001	56 (45·5)	60 (71·4)	93 (70·5)	<0·001	22 (15·0)	55 (38·7)	40 (19·5)	49 (17·2)	<0·001
Broad spectrum, N(%)	111 (62·0)	44 (35·5)	41 (27·9)	28 (21·7)		67 (54·5)	24 (28·6)	39 (29·5)		125 (85·0)	87 (61·3)	165 (80·5)	236 (82·8)	
Co-amoxiclav, N(%)	78 (43·6)	33 (26·6)	34 (23·1)	24 (18·6)	<0·001	41 (33·3)	19 (22·6)	26 (19·7)	0·035	98 (66·7)	74 (52·1)	122 (59·5)	199 (69·8)	0·002
Cephalosporins, N(%)	29 (16·2)	8 (6·5)	6 (4·1)	4 (3·1)	<0·001	25 (20·3)	4 (4·8)	13 (9·8)	0·002	23 (15·6)	11 (7·7)	39 (19·0)	34 (11·9)	0·016
Macrolides, N(%)	4 (2·2)	3 (2·4)	/	/	0·056	1 (0·8)	1 (1·2)	/	0·52	4 (2·7)	2 (1·4)	4 (2·0)	3 (1·1)	5667
Fluoroquinolones, N(%)	/	/	1 (0·7)	/		/	/	/		/	/	/	/	
Amoxicillin dosage, Median [IQR], mg/kg/die	50·0 [10·0]	75·0 [25·0]	75·0 [3·7]	75·0 [0·0]	<0·001	57·6 [14·3]	73·0 [9·1]	75·0 [6·9]	<0·001	/	/	/	/	
Co-amoxiclav dosage, Median [IQR], mg/kg/die	50·0 [10·0]	75·0 [25·0]	72·0 [14·8]	75·0 [5·0]	<0·001	56·1 [8·1]	62·1 [23·7]	68·6 [15·0]	<0·001	/	/	/	/	
DOT, Median [IQR], 2-23 months	8·0 [2·3]	10·0 [2·0]	10·0 [0·0]	10·0 [0·0]	<0·001	10·0 [2·0]	10·0 [0·0]	10·0 [2·0]	0·122	7·0 [1·0]	7·0 [2·0]	7·0 [1·0]	7·0 [1·0]	0·472
DOT, Median [IQR], ≥ 24 months	8·0 [1·5]	7·0 [3·0]	7·0 [3·0]	5·0 [2·0]	<0·001	10·0 [2·0]	7·0 [2·8]	8·0 [5·0]	<0·001	7·0 [1·0]	7·0 [1·0]	7·0 [1·0]	7·0 [1·0]	0·238

Figures

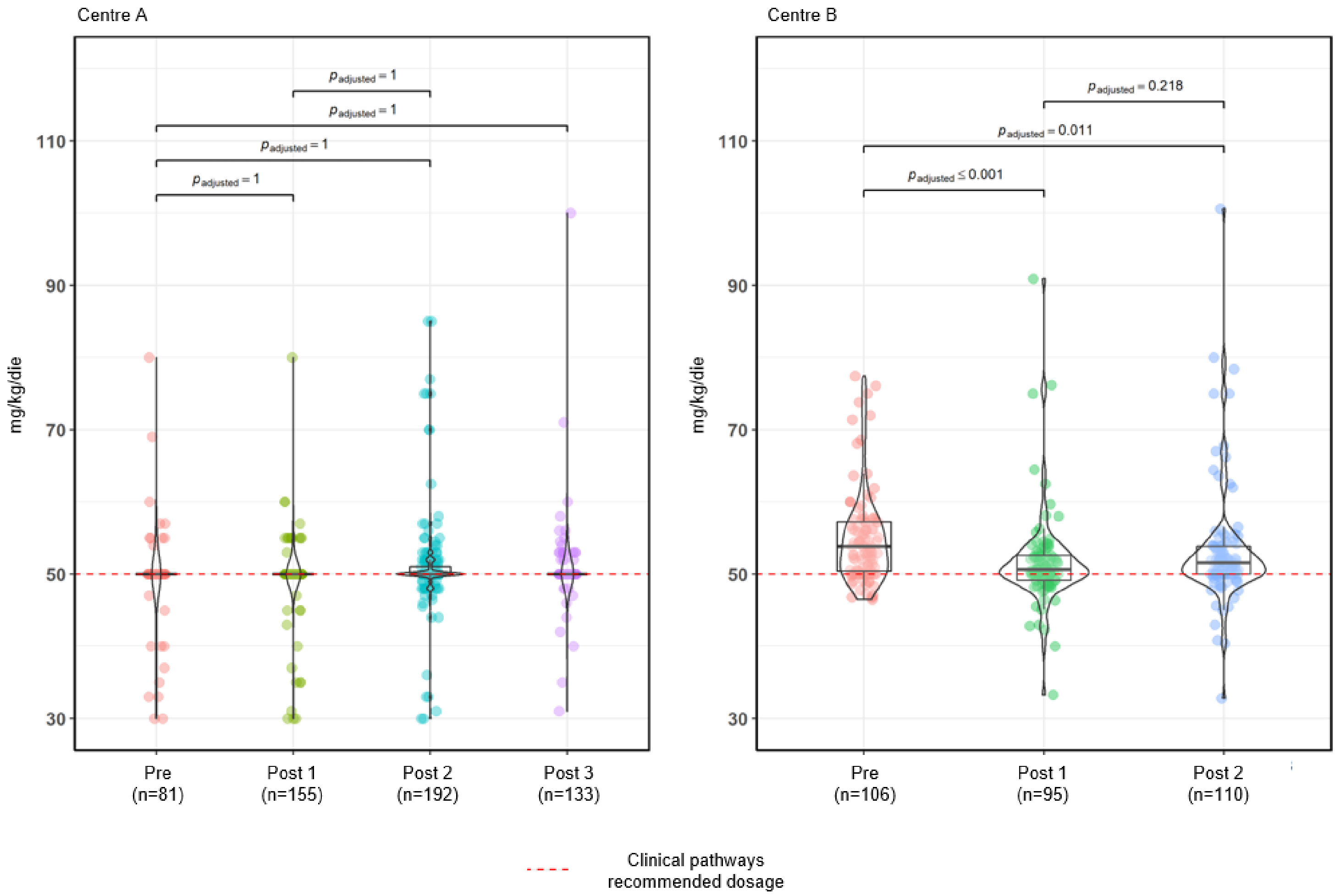

Figure S1. Distribution of amoxicillin dosage for pharyngitis among different periods in Centre A and B with pairwise comparison· The dots represent the granular data, horizontal lines are median and IQR; whiskers extend to the minimum and maximum within 1·5 times the IQR· Violin plots present quantifications. The dotted red line represents the clinical pathways recommended dosage (50 mg/kg/die).

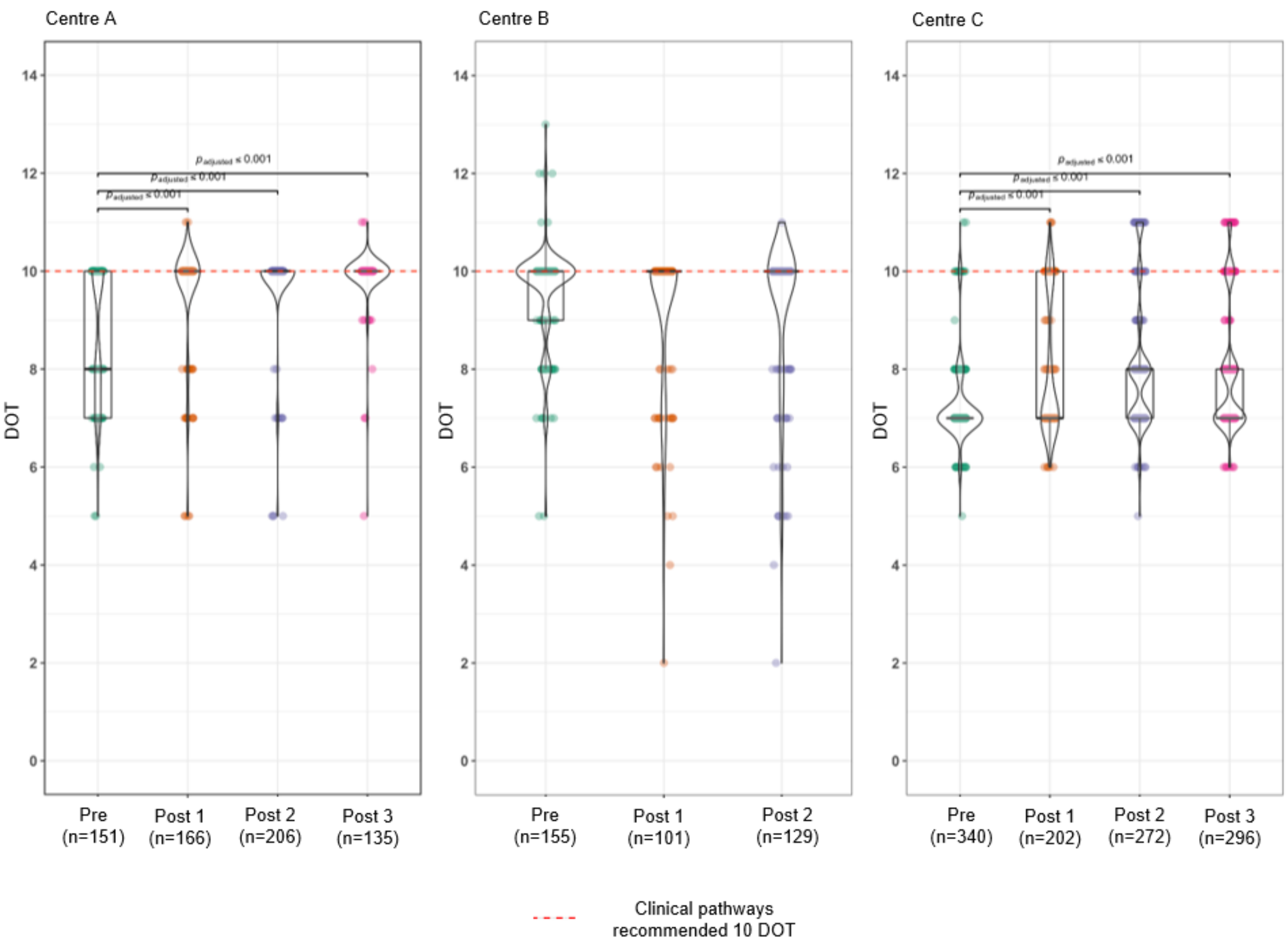

Figure S2 Distribution of days of therapy for pharyngitis among different periods in the three Centers (A, B, C) with pairwise comparison. The dots represent the granular data, horizontal lines are median and IQR; whiskers extend to the minimum and maximum within 1·5 times the IQR. Violin plots present quantifications. The dotted red line represents the clinical pathways recommended 10 DOT.

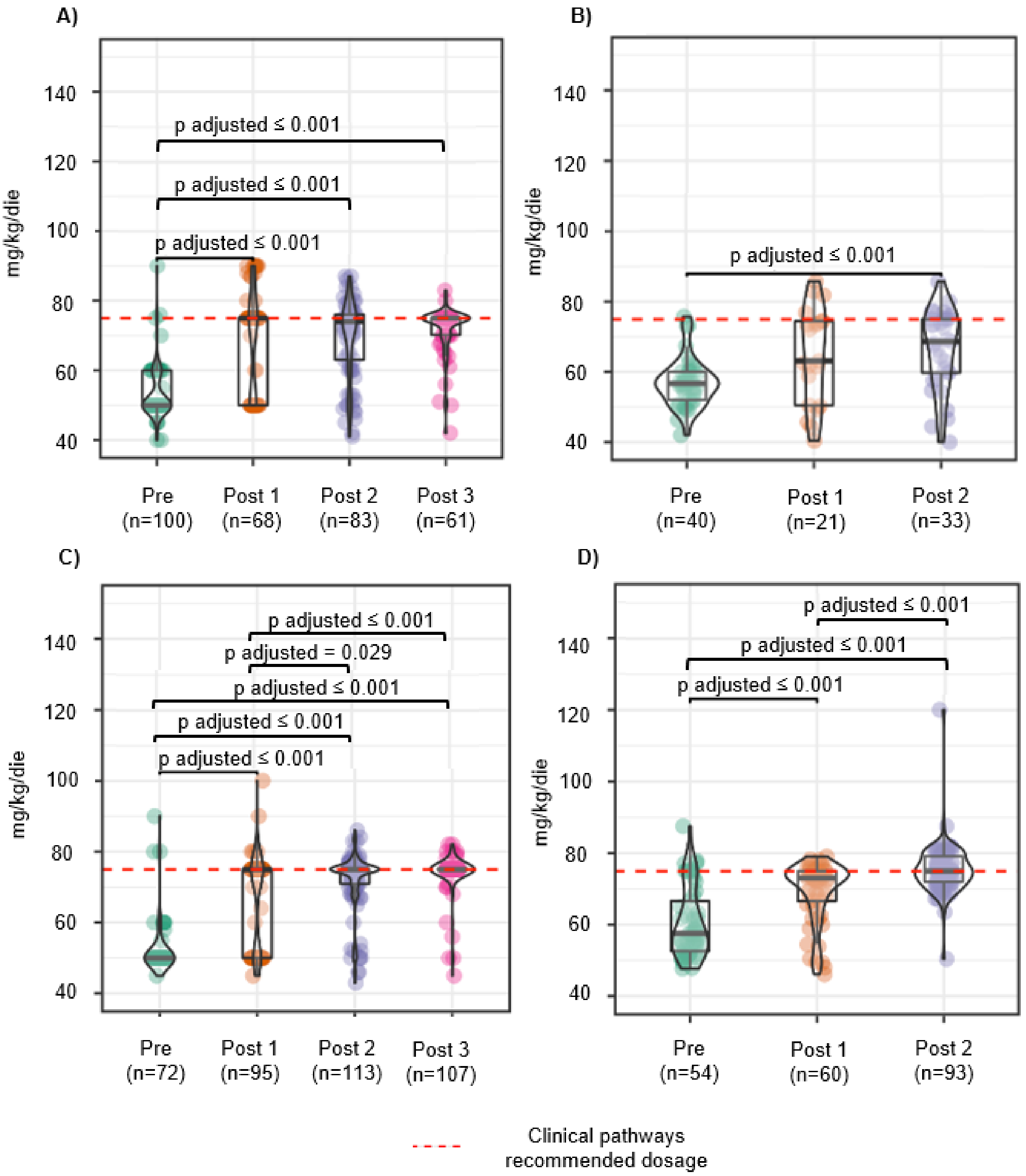

Figure S3. Distribution of co-amoxiclav (A, B) and amoxicillin (C, D) dosage for acute otitis media among different periods in Centre A (A, C) and Centre B (B, D) with pairwise comparison. The dots represent the granular data, horizontal lines are median and IQR; whiskers extend to the minimum and maximum within 1·5 times the IQR. Violin plots present quantifications. The dotted red line represents the clinical pathways recommended dosage (75 mg/kg/die).

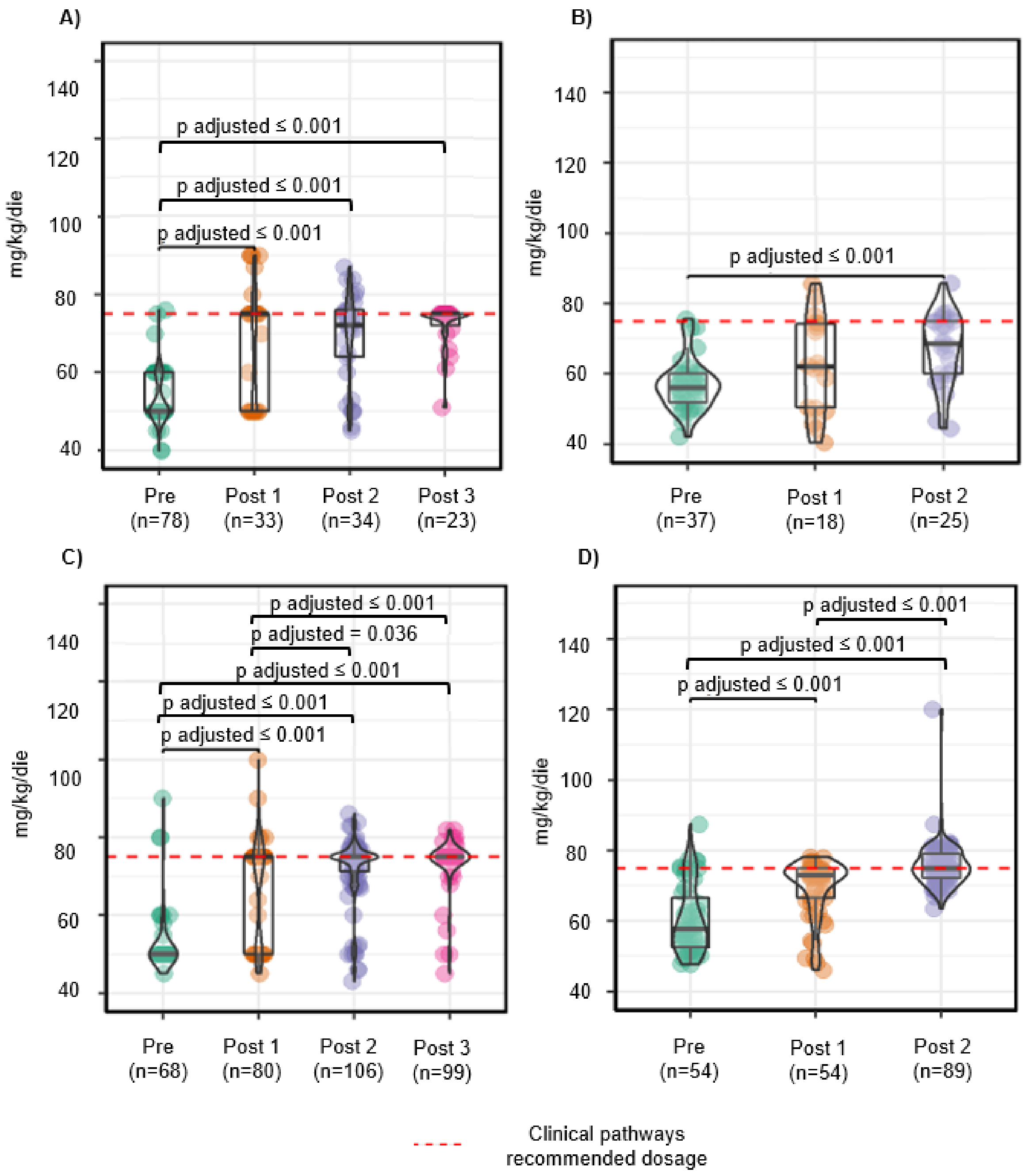

Figure S4. Distribution of co-amoxiclav (A, B) and amoxicillin (C, D) dosage for non- complicated acute otitis media among different periods in Centre A (A, C) and Centre B (B, D) with pairwise comparison. The dots represent the granular data, horizontal lines are median and IQR; whiskers extend to the minimum and maximum within 1·5 times the IQR. Violin plots present quantifications. The dotted red line represents the clinical pathways recommended dosage (75 mg/kg/die).

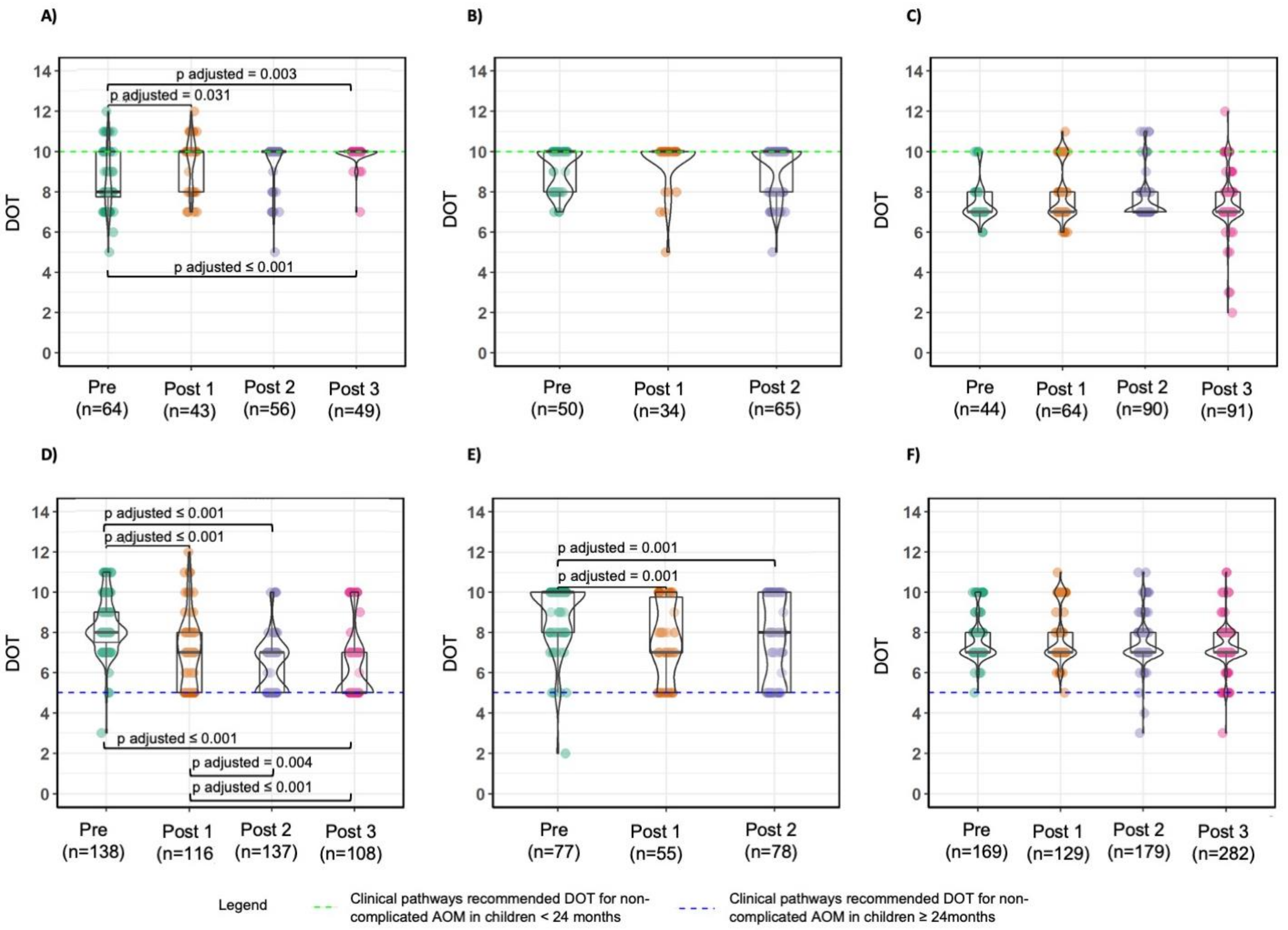

Figure S5 Distribution of days of therapy (DOT) for acute otitis media in the different periods in Centre A (A, D), Centre B (B, E) and Centre C (C, F) stratified by age class (< 24 months: A, B, C; ≥ 24 months: D, E, F) with pair wise comparison. The dots represent the granular data, horizontal lines are median and IQR; whiskers extend to the minimum and maximum within 1·5 times the IQR. Violin plots present quantifications. The dotted green line represents clinical pathways recommended DOT for AOM in children < 24 months while the dotted blue line represents clinical pathways recommended DOT for complicated and non-complicated AOM in children >24 months.
